# Mining biology for antibiotic discovery

**DOI:** 10.1371/journal.pbio.3002946

**Published:** 2024-11-26

**Authors:** Cesar de la Fuente-Nunez

**Affiliations:** 1 Machine Biology Group, Departments of Psychiatry and Microbiology, Institute for Biomedical Informatics, Institute for Translational Medicine and Therapeutics, Perelman School of Medicine, University of Pennsylvania, Philadelphia, Pennsylvania, United States of America; 2 Departments of Bioengineering and Chemical and Biomolecular Engineering, School of Engineering and Applied Science, University of Pennsylvania, Philadelphia, Pennsylvania, United States of America; 3 Department of Chemistry, School of Arts and Sciences, University of Pennsylvania, Philadelphia, Pennsylvania, United States of America; 4 Penn Institute for Computational Science, University of Pennsylvania, Philadelphia, Pennsylvania, United States of America

## Abstract

The rise of antibiotic resistance calls for innovative solutions. This Perspective outlines how biology can be mined digitally using artificial intelligence as a new paradigm for antibiotic discovery, offering hope in the fight against superbugs.

## Teaser

The rise of antibiotic resistance calls for innovative solutions. The realization that biology can be mined digitally using artificial intelligence has revealed a new paradigm for antibiotic discovery, offering hope in the fight against superbugs.

Antibiotic resistance stands as one of the most pressing challenges in modern medicine, transforming once-treatable infections into life-threatening conditions. Despite the desperate need for new antibiotics, traditional drug discovery approaches have failed to keep pace with the rise of resistant pathogens. The discovery of penicillin in the early 20^th^ century revolutionized healthcare, yet the rate of new antibiotic discovery has drastically slowed after an initial growth phase, and is unable to meet the growing demand for novel treatments.

Historically, antibiotic discovery has relied on labor-intensive screening of natural compounds from sources like soil bacteria, fungi, and plants. This approach led to life-saving antibiotics, including penicillin and streptomycin. However, over the past several decades, this method has become increasingly inefficient. The manual nature of the search, the limited diversity of accessible natural compounds, and the rapid emergence of resistance in pathogens have significantly hindered progress.

Several years ago, we proposed an alternative strategy [1]: instead of physically mining biological sources, we could digitally explore the vast repositories of biological data—genomes, metagenomes, and proteomes—that have been accumulated over decades. We hypothesized that such an approach could help accelerate antibiotic discovery by enabling the identification of new molecules at digital speed, rather than through time-consuming manual experiments. Recent advances in artificial intelligence (AI) and computing power are bringing this vision to life. By integrating genomics, machine learning, and computational biology, we can now explore biological data on an unprecedented scale, uncovering hidden antimicrobial agents within a myriad of genomes—including those of both living and extinct organisms. Some of these advances have been made possible through the enhanced capabilities of graphics processing units (GPUs) [2] as well as the development of standardized experimental data sets to train AI models, such as APEX [3], which predicts the antimicrobial activity of specific amino acid sequences. Many of the molecules identified thus far in biological mining efforts have been peptides, many of which are compositionally and physicochemically different from conventional antimicrobial peptides. While other models have focused on small molecules, their application can be potentially constrained by challenges in synthesizability. Nevertheless, AI models facilitate the exploration of previously uncharted sequence spaces in ways that would be infeasible through experimental methods alone.

One significant advance in this field has been the exploration of previously unrecognized types of peptides with antimicrobial activity [3–7]. These include encrypted peptides (EPs)—small peptides hidden within larger proteins that have antimicrobial properties. Early work performed a systematic screen of the entire human proteome, yielding thousands of previously unrecognized EPs [4]. This work involved scaling up mining efforts from one protein at a time to 42,000 proteins, as this exploration went beyond the 20,000 protein-encoding genes of the human genome in search of new antibiotic molecules. This search has since been extended beyond modern humans by digitally identifying molecules from extinct organisms, introducing the field of molecular de-extinction [3,5]. Mining the genomes and proteomes of species like Neanderthals and woolly mammoths has led to the discovery of antimicrobial agents, such as neanderthalin and mammuthusin, the latter of which was identified by the state-of-the-art deep learning model APEX [3]. This new AI model searched through the genetic information of hundreds of extinct organisms and identified over 37,000 potential antibiotics. These findings offer insights into ancient strategies for combating contemporary infections and potentially shed light on the evolution of immunity and infectious diseases.

The potential of digital mining extends beyond higher organisms, reaching into other branches of the tree of life, including the vast and largely uncharacterized diversity of microbial life [6,7]. Much of this microbial “dark matter” remains unexplored, but machine-driven approaches can now analyze microbial genomes and metagenomes to rapidly uncover new antimicrobial molecules. For example, recent computational studies of tens of thousands of microbial genomes and metagenomes have identified nearly 1 million potential antibiotic candidates within the global microbiome [6]. These sequences have been made freely accessible to the scientific community, with the goal of fostering global collaboration and accelerating the development of new antibiotics. Microbes present within the human microbiome also represent a largely untapped frontier in the search for novel antibiotics [7]. By computationally mining data from nearly 2,000 human microbiomes, hundreds of previously unknown antimicrobial molecules have been identified. For instance, prevotellin-2, a compound produced by the gut bacterium *Prevotella copri*, has emerged as a promising candidate in preclinical mouse infection models [7].

Other major recent advances in AI-driven antibiotic discovery include studies that incorporate explainability [8] and identify compounds against metabolically dormant bacteria [9], which are traditionally difficult to eliminate with classic antibiotics.

**Fig 1:**
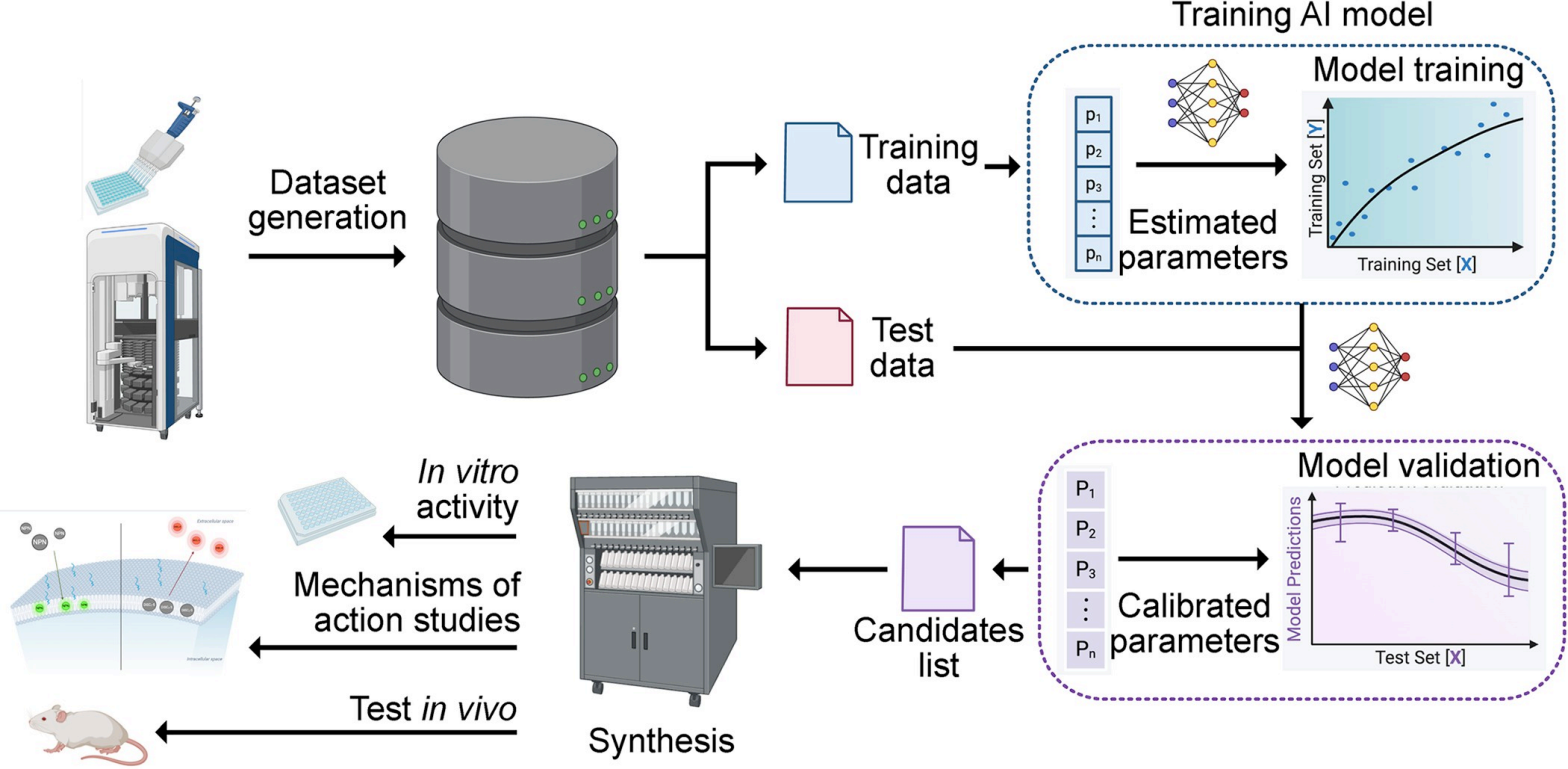
Schematic representation of an AI-assisted antibiotic discovery pipeline. This figure outlines the stages where AI contributes to antibiotic discovery, from data collection to candidate optimization both in vitro and in vivo. Each phase in the pipeline is illustrated with hypothetical candidate compounds identified through AI-driven methods. Created with BioRender.com.

Overall, progress over the past half-decade has accelerated antibiotic discovery, dramatically reducing the time it takes to identify promising candidates—from years to hours [10,11]. Once identified, these peptides can be synthesized and rapidly tested in the lab. This approach has already led to the discovery of many new peptide antibiotics with excellent safety and efficacy profiles in preclinical mouse models (Fig 1).

Despite its promise, this emerging research area faces challenges moving forward. One major hurdle is predicting the safety and efficacy of these molecules in murine infection models and, eventually, in humans. While computational predictions can guide discovery, they must still be validated through rigorous laboratory testing. Additionally, data quality is critical; incomplete or biased data sets could limit the success of AI models, underscoring the need to improve data set quality [12]. Nevertheless, the benefits of digitally mining biology are substantial. Traditional drug discovery pipelines are slow, costly, and often fall short in addressing rising antibiotic resistance. AI-driven approaches offer a faster, more efficient path to discovery.

In conclusion, digital mining of biological data represents a new approach in the search for antibiotics. Leveraging AI with vast, high-quality data sets, could expedite the identification of novel antimicrobial molecules, providing a much-needed tool in the fight against drug-resistant infections. I believe the future of antibiotic discovery will depend on continued advances in AI, automation, and data quality. This future will be inherently transdisciplinary, fostering collaboration between computational scientists and experimentalists. With these challenges addressed, the digital age could usher in a new era of antibiotic discovery, safeguarding global health for generations to come.
